# Investigating the relationship between vasovagal syncope and migraine: a case-control study among Lebanese patients

**DOI:** 10.22514/jofph.2025.047

**Published:** 2025-09-12

**Authors:** Maryline Aouad, Jean Daher, Rita Boulos, Nicole Tannous, Yara Yahchouchi, Souheil Hallit, Kamal Kallab

**Affiliations:** ^1^School of Medicine and Medical Sciences, Holy Spirit University of Kaslik, Jounieh, Lebanon; ^2^Department of Psychology, College of Humanities, Effat University, 21478 Jeddah, Saudi Arabia; ^3^Applied Science Research Center, Applied Science Private University, 11937 Amman, Jordan; ^4^Department of Neurology, Notre Dame des Secours University Hospital Center, Byblos, Lebanon

**Keywords:** Migraine, Vasovagal syncope, Autonomic dysfunction

## Abstract

**Background**: This study investigates the association between migraines and vasovagal syncope (VVS), focusing on shared autonomic dysfunction mechanisms in a Middle Eastern population. **Methods**: Using a case-control design, 163 participants (57 with migraines and 106 controls) were assessed through validated tools, including the Migraine Disability Assessment (MIDAS) and Patient Health Questionnaire-4 (PHQ-4). **Results**: Multivariable analyses results demonstrated a significant association between migraines and VVS, with migraine patients exhibiting nearly threefold higher odds of VVS. Gender differences were pronounced, with females displaying higher susceptibility. Additional drug use and comorbid conditions were also significant factors. Interestingly, the MIDAS score was not found to be significantly correlated with VVS, highlighting the intricacy of autonomic dysfunction. **Conclusions**: These findings underline that screening for autonomic dysfunction must be conducted in migraine patients and that gender-specific approaches should be adopted in their clinical management. Further research is needed to study the causative pathways and to validate these findings in a large and varied population.

## 1. Background

Migraine is a complex neurological condition that affects over 16 % of individuals aged 15 to 39 around the world. It is characterized by the recurrence of episodes of moderate to severe headache, which might be accompanied by nausea, photophobia and phonophobia [1]. According to the International Classification of Headache Disorders (ICHD-3), migraine significantly decreases the quality of life, productivity, and overall well-being [2]. The pathophysiology of migraine does indeed involve the autonomic nervous system (ANS), with evidence of disrupted sympathetic signaling during an attack and impaired cardiovagal function even between episodes [3]. This interplay contributes to a state of autonomic instability wherein the balance of sympathetic and parasympathetic activity is disrupted, leading to chronic and episodic symptoms [4]. Such autonomic dysregulation has been observed in migraine patients, where impaired parasympathetic and sympathetic function were noted in various studies during the interictal period [3, 4, 5]. This widens the view of ANS dysfunction in the pathogenesis of migraine.

Vasovagal syncope (VVS), on the other hand, is the most common cause of transient loss of consciousness due to reflex-mediated hypotension and bradycardia [6]. VVS accounts for up to 35% of first syncope episodes and imposes considerable clinical and economic burdens due to its unpredictability and recurrence [7]. Its etiology is rooted in an exaggerated autonomic response, particularly heightened vagal tone, leading to cardiovascular instability [3, 8]. While migraines and VVS both involve autonomic dysfunction, the manifestations differ: migraines are driven by heightened sympathetic activity during attacks, whereas VVS stems from exaggerated parasympathetic activation, leading to acute hypotension and syncope [9]. These differences highlight distinct downstream effects of shared autonomic pathways. Premonitory symptoms, such as nausea, dizziness, and lightheadedness, further underscore the overlap in clinical presentations [10].

Emerging evidence suggests a potential link between migraines and VVS, with shared mechanisms involving autonomic dysregulation. Studies have identified overlapping features, such as orthostatic intolerance, postural tachycardia syndrome and reflex-mediated syncope, which are significantly more prevalent in migraine patients compared to controls [10, 11]. Furthermore, migraineurs are reported to have higher rates of vagal hyperactivity, which may predispose them to cardiovascular instability, as seen in VVS [10]. Despite these findings, the exact mechanisms by which these similarities contribute to distinct outcomes—chronic pain in migraines versus transient loss of consciousness in VVS—remain poorly understood [9]. Genetic factors may also play a role, as suggested by familial studies linking migraines and syncope through shared vascular dysregulation pathways [12].

Understanding this association is crucial for several reasons. First, identifying shared mechanisms could provide insights into novel diagnostic and therapeutic approaches targeting autonomic dysfunction. For instance, targeting vagal hyperactivity might improve management strategies for both conditions. Second, investigating this relationship in Middle Eastern populations, where autonomic dysfunction in migraines and VVS remains understudied, could contribute region-specific insights into clinical care. Finally, exploring demographic and clinical modifiers, including gender, age, anxiety, depression, and disease severity, may help identify subgroups at higher risk for coexisting conditions. This study seeks to bridge these gaps by investigating the prevalence of VVS in individuals with migraines and elucidating the role of vagal hypertonia, with implications for clinical practice and patient management.

## 2. Materials and methods

This study employed a case-control design to investigate the relationship between migraines and VVS among Lebanese patients. A total of 163 participants were recruited, including 57 individuals diagnosed with migraines (case group) and 106 matched controls without migraines (control group). Control participants were selected conveniently from the same population as cases, without strict matching for age. The age range of the control group (15–70 years) slightly exceeded that of the migraine group (15–60 years), reflecting availability constraints during recruitment. However, gender matching was maintained. Given the available sample size, an approximate 1:2 ratio was achieved where possible, without the use of propensity score matching. This matching technique helped minimize confounding. Data collection was conducted in December 2024 after obtaining ethical approval from the Institutional Ethics Committee.

On the other hand, participants were included in the migraine group if they were adults aged 15–60 years with a confirmed diagnosis of migraines according to the International Classification of Headache Disorders (ICHD-3). The control group included adults aged 15–60 years without a history of migraines or other significant neurological disorders. General inclusion criteria required participants to provide informed consent, complete the study questionnaires, and have no contraindications for autonomic testing. Participants were excluded if they had major cardiovascular, respiratory, or neurological conditions; were pregnant; or were using medications significantly affecting autonomic function (*e.g.*, beta-blockers).

Data for this study were collected using a structured 31-question survey consisting of three main sections: demographics, the Migraine Disability Assessment (MIDAS) scale and the Patient Health Questionnaire-4 (PHQ-4). The demographics section captured essential participant characteristics, including age, gender, smoking habits, alcohol consumption, comorbidities, and chronic medication use. The MIDAS scale, a validated tool designed to measure the impact of migraines on daily activities over the past three months, was used to assess migraine-related disability. Scores were categorized into no/mild, mild, moderate, and severe disability based on established thresholds. The MIDAS scale has been extensively validated in various settings, including studies examining its reliability in assessing work-related disability among chronic migraine sufferers [13] and its utility in cross-cultural contexts, such as in Arabic-speaking populations [14].

The PHQ-4, a brief screening tool for anxiety and depression, was included to evaluate psychological distress. Participants rated four items on a 4-point Likert scale, with total scores ranging from 0 to 12. Anxiety and depression sub-scores were also calculated. This tool has been validated in multiple studies, including its application in general population mental health screening [15] and in identifying psychological comorbidities in patients with chronic illnesses [16]. The inclusion of these validated instruments in this study ensures the reliability and accuracy of data collection, particularly in assessing migraine-related disability and psychological distress.

The diagnosis of VVS in this study was clinically done based on a detailed history and symptomatology rather than a validated diagnostic tool. The questionnaire was specifically designed for this purpose by a group of specialists. Key diagnostic criteria included a history of transient loss of consciousness associated with typical premonitory symptoms, such as dizziness, nausea and sweating, and rapid recovery with no neurological sequelae. Although no standardized questionnaire was used, the clinical diagnosis was based on established criteria for the diagnosis of reflex-mediated syncope; therefore, maintaining reliability and consistency across cases. Although not formally validated, this questionnaire has been developed from the established clinical guidelines on reflex-mediated syncope and was tested for accuracy and internal consistency by a panel of experts in the field. This was done to ensure a systematic and comprehensive assessment of participants for VVS, as there is no population-specific validated diagnostic tool.

Data analysis was performed using SPSS version 27 (IBM Corp., Armonk, NY, USA). Continuous variables were tested for normality using skewness and kurtosis. Normally distributed variables were analyzed using Student’s *t*-test, while non-normally distributed variables were analyzed using the Mann-Whitney U test. Categorical variables were analyzed using Chi-square or Fisher’s exact test. Logistic regression was conducted to determine factors associated with VVS, with VVS (yes/no) as the dependent variable. Independent variables with *p*-values < 0.25 in bivariate analyses were included in the regression model. Statistical significance was set at *p* < 0.05.

## 3. Results

A total of 163 participants completed the questionnaire, with the majority aged between 18–24 years (37.4%) and 72.4% females. All participant characteristics can be found in Table 1.

**Table 1.** Sociodemographic and other characteristics of participants (N = 163).

Characteristics		N (%)
Age (yr)		
	15–18	13 (8.0%)
	18–24	61 (37.4%)
	25–34	26 (16.0%)
	35–44	17 (10.4%)
	45–54	25 (15.3%)
	55 and above	21 (12.9%)
Sex		
	Male	45 (27.6%)
	Female	118 (72.4%)
Smoking		
	None	96 (58.9%)
	Occasionally	22 (13.5%)
	Weekly	12 (7.4%)
	Daily	33 (20.2%)
Alcohol drinking		
	None	45 (27.6%)
	Occasionally	102 (62.6%)
	Weekly	14 (8.6%)
	Daily	2 (1.2%)
Additional drugs		
	No	113 (70.2%)
	Yes	48 (29.8%)
Migraine		
	No	106 (65.0%)
	Yes	57 (35.0%)
Hypertension		
	No	143 (87.7%)
	Yes	20 (12.3%)
Cardiac problems		
	No	154 (94.5%)
	Yes	9 (5.5%)
Dyslipidemia		
	No	133 (81.6%)
	Yes	30 (18.4%)
Diabetes		
	No	158 (96.9%)
	Yes	5 (3.1%)
Epilepsy		
	No	159 (97.5%)
	Yes	4 (2.5%)
Other diseases		
	No	143 (87.7%)
	Yes	20 (12.3%)
Vasovagal syncope		
	No	140 (85.9%)
	Yes	23 (14.1%)
		Mean ± SD
MIDAS total		18.93 ± 52.26
PHQ-4 total		3.76 ± 2.99

*MIDAS: Migraine Disability Assessment; PHQ-4: Patient Health Questionnaire-4; SD: Standard Deviation.*

Fig. 1 illustrates the distribution of PHQ-4 scores in participants with and without VVS. Although the median PHQ-4 score was slightly higher in the VVS group, the overlap in interquartile ranges suggests that anxiety and depression symptoms, as measured by PHQ-4, were not markedly different between groups. This aligns with the lack of statistical significance observed in the bivariate analysis for PHQ-4 scores.

**Fig. 1.** Box plot of PHQ-4 scores by VVS group. PHQ-4: Patient Health Questionnaire-4; VVS: vasovagal syncope.

### 3.1 Bivariate analysis of factors associated with vasovagal syncope

A higher percentage of females (95.7% *vs.* 68.6%; *p *= 0.007), of patients who take additional drugs (56.5% *vs.* 25.4%; *p* = 0.002), who have migraine (65.2% *vs.* 30.0%; *p* = 0.001) and other diseases (26.1% *vs.* 10.0%; *p* = 0.041) was significantly found in the group who had vasovagal syncope (Table 2).

**Table 2.** Bivariate analyses of factors associated with vasovagal syncope.

Variable		No vasovagal syncope	Vasovagal syncope	*p*	Effect size
Age (yr)					
	15–18	12 (8.6%)	1 (4.3%)	0.807	0.119
	18–24	52 (37.1%)	9 (39.1%)		
	25–34	21 (15.0%)	5 (21.7%)		
	35–44	15 (10.7%)	2 (8.7%)		
	45–54	23 (16.4%)	2 (8.7%)		
	55 and above	17 (12.1%)	4 (17.4%)		
Sex					
	Male	44 (31.4%)	1 (4.3%)	**0.007**	0.211
	Female	96 (68.6%)	22 (95.7%)		
Smoking					
	None	82 (58.6%)	14 (60.9%)	0.860	0.068
	Occasionally	18 (12.9%)	4 (17.4%)		
	Weekly	11 (7.9%)	1 (4.3%)		
	Daily	29 (20.7%)	4 (17.4%)		
Alcohol drinking					
	None	39 (27.9%)	6 (26.1%)	0.434	0.130
	Occasionally	87 (62.1%)	15 (65.2%)		
	Weekly	13 (9.3%)	1 (4.3%)		
	Daily	1 (0.7%)	1 (4.3%)		
Additional drugs					
	No	103 (74.6%)	10 (43.5%)	**0.002**	0.238
	Yes	35 (25.4%)	13 (56.5%)		
Migraine					
	No	98 (70.0%)	8 (34.8%)	**0.001**	0.257
	Yes	42 (30.0%)	15 (65.2%)		
Hypertension					
	No	121 (86.4%)	22 (95.7%)	0.313	0.098
	Yes	19 (13.6%)	1 (4.3%)		
Cardiac problems					
	No	133 (95.0%)	21 (91.3%)	0.472	0.056
	Yes	7 (5.0%)	2 (8.7%)		
Dyslipidemia					
	No	114 (81.4%)	19 (82.6%)	1.000	0.011
	Yes	26 (18.6%)	4 (17.4%)		
Diabetes					
	No	135 (96.4%)	23 (100.0%)	1.000	0.072
	Yes	5 (3.6%)	0 (0.0%)		
Epilepsy					
	No	136 (97.1%)	23 (100.0%)	1.000	0.064
	Yes	4 (2.9%)	0 (0.0%)		
Other diseases					
	No	126 (90.0%)	17 (73.9%)	**0.041**	0.171
	Yes	14 (10.0%)	6 (26.1%)		
MIDAS total		15.86 ± 50.94	37.61 ± 57.37	**<0.001**	0.419
PHQ-4 total		3.64 ± 2.76	4.48 ± 4.12	0.357	0.280

*Numbers in bold indicate significant p values. MIDAS: Migraine Disability Assessment; PHQ-4: Patient Health Questionnaire-4.*

Fig. 2 presents the distribution of MIDAS scores among participants with and without VVS. Individuals with VVS exhibited significantly higher MIDAS scores compared to those without VVS, indicating greater migraine-related disability in this group. The median MIDAS score was substantially elevated in the VVS group, with a broader interquartile range, reflecting the variability in migraine-related disability among these individuals.

**Fig. 2.** Box plot of MIDAS Scores by VVS group. MIDAS: Migraine Disability Assessment; VVS: vasovagal syncope.

### 3.2 Multivariate analysis of factors associated with vasovagal syncope

The results of logistic regression, taking vasovagal syncope as the dependent variable, showed that females compared to males (adjusted odds ratio (aOR) = 9.28), having migraine (aOR = 2.99) and taking additional drugs (aOR = 2.96) were significantly associated with higher odds of having vasovagal syncope (Table 3, Model 1). When taking the MIDAS score as an independent variable, the results showed that the MIDAS score was not significantly associated with vasovagal syncope (Table 3, Model 2).

**Table 3.** Logistic regression taking vasovagal syncope (Yes/No) as the dependent variable.

Variables		*p*	aOR	95% CI
Model 1: migraine (yes/no) as an independent variable (Nagelkerke *R*^2^ = 0.242)				
	Gender (females *vs.* males*)	**0.036**	9.28	1.16–74.43
	Migraine (Yes *vs.* No*)	**0.030**	2.99	1.11–8.06
	Additional drugs (Yes *vs.* No*)	**0.031**	2.96	1.10–7.93
	Other diseases (Yes *vs.* No*)	0.382	1.70	0.52–5.57
Model 2: MIDAS score as an independent variable (Nagelkerke *R*^2^ = 0.221)				
	Gender (females *vs.* males*)	**0.034**	9.35	1.18–73.98
	MIDAS score	0.08	1.01	0.99–1.01
	Additional drugs (Yes *vs.* No*)	**0.007**	3.85	1.46–10.19
	Other diseases (Yes *vs.* No*)	0.254	1.97	0.61–6.34

*Reference group. Numbers in bold indicate significant p values. MIDAS: Migraine Disability Assessment; aOR: adjusted odds ratio; CI: Confidence Interval.

Fig. 3 is a forest plot that illustrates the results of logistic regression analyses, displaying the adjusted odds ratios (aOR) with 95% confidence intervals for factors associated with vasovagal syncope. Significant associations are marked in red. Gender (females *vs.* males) demonstrated the strongest association with vasovagal syncope (aOR = 9.28, *p* = 0.036), followed by having migraines (aOR = 2.99, *p* = 0.030) and the use of additional drugs (aOR = 2.96, *p* = 0.031). Other diseases did not show a significant association with VVS (aOR = 1.70, *p* = 0.382). The confidence intervals for significant variables do not cross the reference line (OR = 1), confirming their significance.

**Fig. 3.** Forest plot depicting adjusted odds ratios for variables associated with VVS.

### 3.3 Stratification by age categories and gender

When stratifying the results by age categories and gender, the results showed that a higher percentage of patients of females (63.6% *vs.* 33.3%; *p* = 0.009), and those who had migraine in the 25–34 years (100% *vs.* 33.3%; *p* = 0.012) and in the 55+ years category (100% *vs.* 17.6%; *p* = 0.006) had vasovagal syncope (Table 4).

**Table 4.** Association of migraine and vasovagal syncope.

Stratification group	Migraine	No vasovagal syncope	Vasovagal syncope	*p*	Effect size
15–18 yr					
	No	11 (91.7%)	0 (0.0%)	0.154	0.677
	Yes	1 (8.3%)	1 (100.0%)		
18–24 yr					
	No	43 (82.7%)	5 (55.6%)	0.087	0.235
	Yes	9 (17.3%)	4 (44.4%)		
25–34 yr					
	No	14 (66.7%)	0 (0.0%)	**0.012**	0.527
	Yes	7 (33.3%)	5 (100.0%)		
35–44 yr					
	No	5 (33.3%)	1 (50.0%)	1.000	0.112
	Yes	10 (66.7%)	1 (50.0%)		
45–54 yr					
	No	11 (47.8%)	2 (100.0%)	0.480	0.283
	Yes	12 (52.2%)	0 (0.0%)		
55 yr and above					
	No	14 (82.4%)	0 (0.0%)	**0.006**	0.686
	Yes	3 (17.6%)	4 (100.0%)		
Males					
	No	34 (77.3%)	0 (0.0%)	0.244	0.265
	Yes	10 (22.7%)	1 (100.0%)		
Females					
	No	64 (66.7%)	8 (36.4%)	**0.009**	0.257
	Yes	32 (33.3%)	14 (63.6%)		

*Numbers in bold indicate significant p values.*

## 4. Discussion

This study found a significant association between migraines and VVS, with migraine patients being nearly three times more likely to experience VVS compared to controls (aOR = 2.99, 95% CI: 1.11–8.06, *p* = 0.030). Gender differences were particularly striking, with females exhibiting a ninefold higher likelihood of VVS compared to males (aOR = 9.28, 95% CI: 1.16–74.43, *p* = 0.036). Additionally, patients taking multiple medications and those with other comorbid conditions were more likely to have VVS. These findings underscore the multifactorial nature of the migraine-VVS relationship.

The association between migraines and VVS can be attributed to shared autonomic dysfunction, specifically involving vagal hypertonia and imbalances in the sympathetic-parasympathetic system. Prior studies [3, 7, 11] have emphasized the role of autonomic dysregulation in both conditions, particularly vagal hyperactivity leading to cardiovascular instability in VVS. Similarly, the CAMERA study [10] demonstrated a higher prevalence of syncope and orthostatic intolerance in migraine patients compared to controls, consistent with the significant association observed in this study. The study [5] further supports the link, identifying vagal hyperactivity and parasympathetic hypoactivity as key mechanisms underlying these conditions.

The pronounced gender differences observed in this study may stem from hormonal influences on autonomic regulation. Estrogen, which fluctuates during the menstrual cycle, has been shown to modulate autonomic tone. Recent findings by [17] indicate that elevated estrogen levels during the luteal phase are associated with increased sympathetic activity, while lower levels in the follicular phase correspond with greater parasympathetic tone. These shifts in autonomic balance may contribute to differing susceptibilities to vasovagal responses across the cycle, with lower estrogen potentially facilitating syncope episodes. The aforementioned physiological patterns may underline, at least in part, the observed gender disparity in VVS incidence. Consistent with our findings, Khurana and Van Meerbeke [18] reported a higher prevalence of syncope-related symptoms among females, further reinforcing the role of gender-specific autonomic mechanisms.

Interestingly, the MIDAS score, while effective for assessing migraine-related disability, did not show a significant association with VVS in this analysis, in other words, higher migraine-related disability did not reflect an underlying susceptibility to vasovagal syncope. Similar to findings by [12], this may reflect the complexity of autonomic dysfunction in migraines, where subjective disability scores may not fully capture physiological autonomic changes. Alternatively, the lack of significance could reflect sample size limitations or variability in MIDAS scores.

The findings of this study have significant clinical implications. The strong association between migraines and VVS underscores the importance of screening migraine patients for symptoms of autonomic dysfunction, particularly VVS, during clinical evaluations. This is particularly critical for female patients, who demonstrated markedly higher odds of VVS, warranting gender-specific approaches to prevention and management. Additionally, the identification of drug use and comorbid conditions as significant factors highlights the need for comprehensive medical histories to guide treatment decisions. Such findings would, therefore, support the idea of interventions directed toward the shared pathophysiological mechanisms-such as autonomic dysfunction-to improve outcomes for patients with migraine and VVS.

## 5. Limitations

However, there are several limitations that should be considered in this study. The cross-sectional design does not allow for any indication of a causal relationship in either direction between migraines and VVS. The sample size is relatively modest and especially in subgroup analyses, the power to detect smaller associations/interactions may be reduced, therefore, future multi-center studies with larger samples would be needed to confirm the trends. Moreover, in diagnosing VVS and filling out the questionnaires, there is reliance on self-reported data that can therefore include recall bias. While this could affect the precision of reported symptoms, it does not compromise the internal reliability of the structured questionnaire used. Additionally, the cases in this study were clinically straightforward, and no further diagnostic tools were deemed necessary. Future studies should consider incorporating externally validated diagnostic instruments, especially when evaluating more complex or ambiguous presentations. In line with this, the control group had a slightly broader age range, which may introduce minor age-related bias. However, this was considered acceptable given the clinical clarity of cases and consistent source population. Although already validated tools like MIDAS and PHQ-4 were used, there is a likelihood that these scales may not fully capture the complexity of autonomic dysfunction. The reliance on self-report questionnaires (MIDAS, PHQ-4) introduces risk of bias and subjective interpretation, particularly in assessing migraine disability and psychological distress. Moreover, the fact that the study was conducted on a Middle Eastern population limits the generalization of such findings to other regions. Future studies should overcome these limitations by using longitudinal designs, larger cohorts, and objective measures of autonomic function.

## 6. Conclusions

The current investigation identifies the critical connection of migraines with VVS. Major predisposing factors have been found to include gender, use of medications and presence of comorbid conditions. These data confirm the contribution of autonomic dysfunction, in particular, vagal hyperactivity and imbalance between parasympathetic and sympathetic drives in the pathophysiology of the disorders under investigation. Gender-specific differences further stress the need for gender-specific clinical approaches, and the lack of association between MIDAS scores and VVS indicates the complexity of autonomic involvement in migraines. Focusing on a population from the Middle East, this study offers new insights into an under-investigated demographic and underscores the importance of comprehensive patient evaluation. Future studies should incorporate causal pathways, increase sample sizes, and include objective measures of autonomic function to better understand these associations and help develop targeted interventions.
